# ADVERSE CHILDHOOD EXPERIENCES AND DEPRESSIVE SYMPTOMS IN MIDDLE-AGED AND OLDER CHINESE ADULTS

**DOI:** 10.1093/geroni/igad104.3046

**Published:** 2023-12-21

**Authors:** Wenxing Wei, Aloen Townsend

**Affiliations:** Case Western Reserve University, Cleveland, Ohio, United States; Case Western Reserve University, Cleveland, Ohio, United States

## Abstract

Prior research has shown a significant effect from adverse childhood experiences (ACEs) to depressive symptoms in later adulthood, but there is very limited research on this relationship in China. This study examines the relationship between multiple childhood adversity indicators (childhood abuse, bullying victimization, and witnessing violence) and depressive symptoms in Chinese middle-aged and older adults by gender. The study was based on a nationally representative sample of 10,371 participants aged 45 and older collected through the China Health and Retirement Longitudinal Study (CHARLS). Childhood adversity indicators in the 2014 Life History Survey were merged with the harmonized 2018 CHARLS dataset. Structural equation modeling was conducted separately for males and females because the measurement model was not invariant by gender. Childhood abuse was not a significant predictor for either gender but bullying victimization and witnessing violence were significant. Witnessing violence was a stronger predictor for females (gamma = .15, p < .001) than for males (gamma = .08, p < .05). For both groups, childhood adversity variables together accounted for 6% variance in depressive symptoms. Experiences of being bullied in school and community and witnessing violence in the family were still influential in middle and late life. Figuring out what role ACEs are playing and how to deal with their effects are vital to providing more effective prevention and interventions. Further investigations are required, for example, regarding the mechanisms behind the significant relationships and why childhood abuse was not a significant predictor among this Chinese sample.

